# Modelling the stand dynamics after a thinning induced partial mortality: A compensatory growth perspective

**DOI:** 10.3389/fpls.2022.1044637

**Published:** 2022-12-07

**Authors:** Chao Li, Hugh Barclay, Shongming Huang, Bernard Roitberg, Robert Lalonde, Wenli Xu, Yingbing Chen

**Affiliations:** ^1^ Canadian Wood Fibre Centre, Canadian Forest Service, Edmonton, AB, Canada; ^2^ Pacific Forestry Centre, Canadian Forest Service, Victoria, BC, Canada; ^3^ Alberta Agriculture, Forestry and Rural Economic Development, Edmonton, AB, Canada; ^4^ Department of Biological Sciences, Simon Fraser University, Burnaby, BC, Canada; ^5^ Department of Biology, University of British Columbia, Okanagan, Canada; ^6^ Ministry of Forests, Lands, Natural Resource Operations and Rural Development, Victoria, BC, Canada

**Keywords:** over-compensation, partial harvest, productivity evaluation, stand growth trajectory, tree-based state-dependent model, TreeCG, Wood Fibre Value Simulation Model

## Abstract

**Introduction:**

With increasing forest areas under management, dynamics of managed stands have gained more attention by forest managers and practitioners. Improved understanding on how trees and forest stands would respond to different disturbances is required to predict the dynamics of managed stand.s. Partial mortality commonly occurs in stand development, and different response patterns of trees and stands to partial mortality would govern stand dynamics.

**Methods:**

To investigate the possible response patterns using existing knowledge of growth and yield relationships, we developed TreeCG model, standing for Tree’s Compensatory Growth, a state-dependent individual tree-based forest growth model that simulates the compensatory growth of trees after experiencing a partial mortality. The mechanism behind the simulation is the redistribution of resources, including nutrients and space, freed from died trees to surviving trees. The developed new algorithm simplified the simulations of annual growth increments of individual trees over a long period of stand development.

**Results:**

The model was able to reproduce the forest growth patterns displayed in long-term precommercial thinning experiments. The simulated forest growth displayed the process of compensatory growth from under compensation, to compensation-induced-equality, and to overcompensation over time.

**Discussion:**

Our model can simulate stand growth trajectories after different partial harvest regimes at different times and intensities, thus support decisions in best partial harvest strategies. This generic model can be refined with given tree species and specific site conditions to predict stand dynamics after given partial mortality for any jurisdictions under management. The simulation reassembles growth trajectories of natural stands when no thinning is conducted.

## Introduction

Forest productivity, defined as the accumulated standing forest volume since stand initiation, and often referred to as yield in studies of growth and yield (Avery and Burkhart, 1994; Weetman and Mitchell, 2013), can be influenced by many factors including changes in environmental conditions such as disturbances and climate change (e.g., Shugart et al., 2003; Shell and Bongers, 2020). To improve forest productivity forecasting, it is crucial to understand the response patterns of trees and forest stands to changing environmental conditions to determine the adaptation strategy in sustainable forest management. Climate change, as one particular change in environmental conditions, can influence forest dynamics either directly or indirectly (Crookston et al., 2010). The direct influence could occur *via* altered growth rates of trees due to changing environmental pressure or by altering the suitability of ecological niche for the species (e.g., Hiura et al., 2019). Such indirect influence could be through changing natural disturbance regimes, which could in turn modify the mortality of trees and thus the stage structure of forest stands (e.g., Dale et al., 2001; IPCC, 2014). The current study focuses on how forest stands might respond to partial mortality, using controllable thinning operations as an example, to reduce stochastic uncertainties in partial mortality caused by natural disturbances.

Partial mortality may be common in forest stand development caused by many different factors including various types of disturbances, such as thinning. Thinning is a partial-cut treatment where a portion of the trees in the stand are harvested, which modifies the subsequent growth process of that stand. It is a silvicultural intervention tool that is used to modify the process of natural stand development for achieving desirable forest conditions, serving as a form of anthropogenic disturbances (e.g., Nyland, 1996; Ares et al., 2009; Kitagawa et al., 2018). When performed before the trees are ready for harvest for commodity products, it is called precommercial thinning (PCT), which reduces stand density and generally results in faster growth of remaining trees in diameter than that of untreated sites (Weetman and Mitchell, 2013). As a result, trees with bigger diameters are expected to favor lumber industries and increase the stand value dramatically when trees grow into the sawlog class.

Although short-term observations seldom demonstrated that stand gross volumes in thinned sites exceed those in unthinned sites (e.g., Bose et al., 2018), several long-term observations confirm that stand gross volumes in many treated sites can exceed that in untreated sites as surveyed by Li et al. (2020). For example, 42 years following initial PCT treatments, balsam fir (*Abies balsamea* (L.) Mill.) stand volumes in treated sites were about 15% higher than those from untreated sites in the Green River watershed of northwestern New Brunswick, Canada (Pitt and Lanteigne, 2008). Harvest analyses raised questions on whether this apparent productivity enhancement might be a common expectation from PCT, and how this could influence the forecast of long-term stand productivity, which is the foundation of sustainable forest management planning (e.g., Manitoba Conservation and Water Stewardship, 2013). To answer these questions, understanding of the mechanism(s) behind the observations becomes a necessity.

Compensatory growth (CG) is a common cross-taxa phenomenon in biology (Li et al., 2021). The earliest report of CG can be traced back to Osborne and Mendel (1915; 1916) in their famous study on nutrition and growth. Though CG has been termed differently in different biological fields, the essential meaning is consistent. For example, Mangel and Munch (2005) referred to CG as the ability of an organism to grow at an accelerated rate following a period of resource deprivation. Diverse CG patterns have been found, and they can be grouped into three main statuses (Belsky, 1986; Maschinski and Whitham, 1989; Whitham et al., 1991; Li et al., 2020; Li et al., 2021):

1. under-compensation, in which biomass or body mass of treated group is lower than that of untreated group;
2. compensation-induced-equality (CIE) or exact compensation, in which biomass or body mass of treated group equals to that of untreated group; and
3. over-compensation, in which biomass or body mass of treated group exceeds that of untreated group.

**Figure 1:**
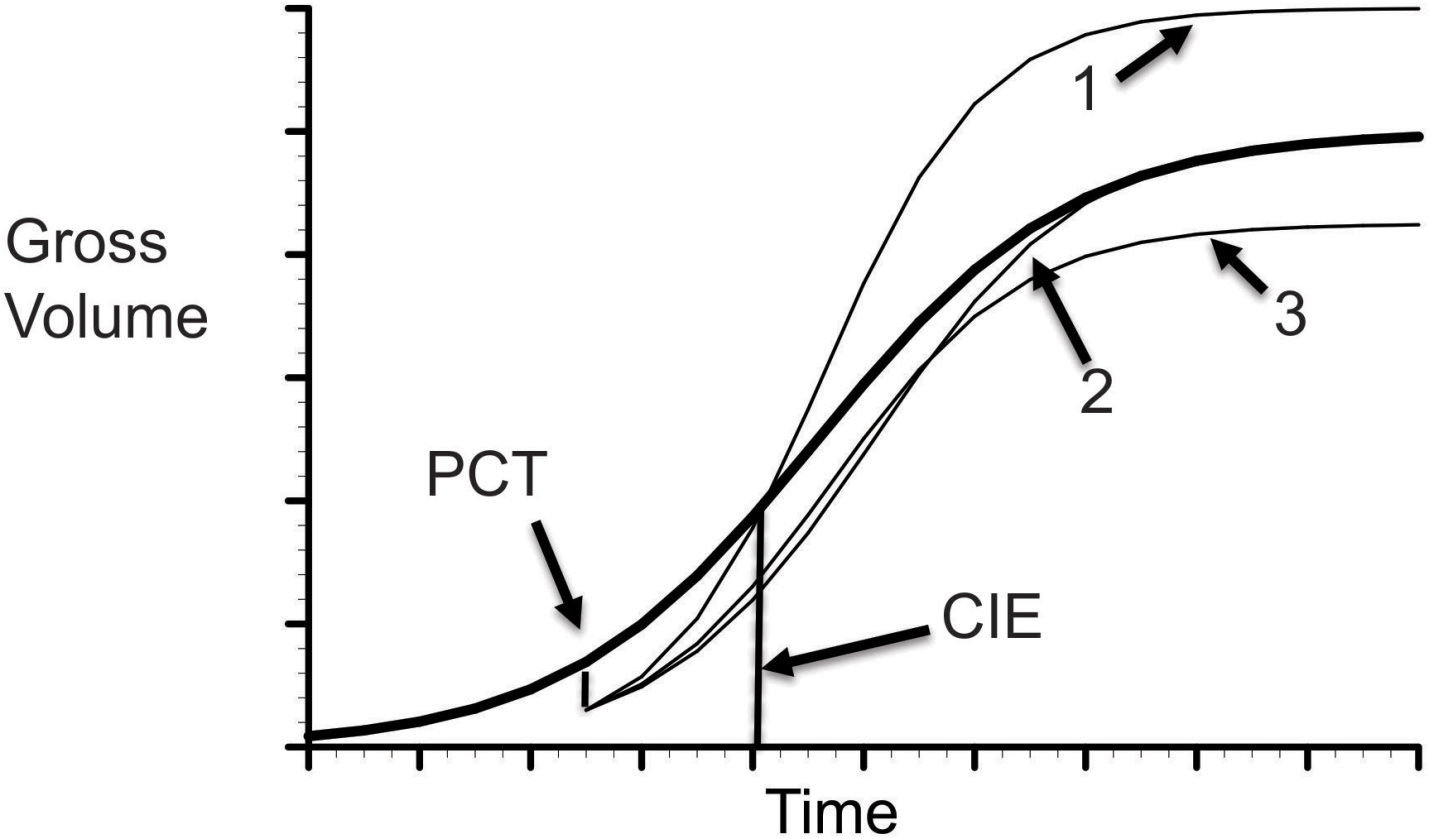
Illustration of CG. The solid line represents gross volume in natural stands. The dished lines indicate possible compensatory outcomes to a PCT operation at 12 years after planting: 1 - over-compensation; 1 - exact compensation, and 3 - under-comensation.

Each of these hypothetical patterns for CG following a PCT are showed in **Figure 1**.

CG has been observed in plants, invertebrates, and vertebrates, both in the laboratory and in the wild and in both juveniles and adults as reviewed in Li et al. (2021). Mangel and Munch (2005) summarized that the diverse CG patterns are dependent on species, social environment, seasonal development, temperature, food availability, and physiological factors such as internal state and age. Therefore, there is no single “fixed” pattern of CG, but there may be general principles that allow deepening understanding and predicting the patterns, given the context.

In forestry, CG can be defined as a change in growth rate, usually positive, in a forest stand following a disturbance that reduces biomass and/or individuals from the population (Li et al., 2021). For example, the lens of CG was able to explain the over-compensation displayed in the coastal Douglas-fir (*Pseudotsuga menziesii* [Mirb.] Franco) stands near the Shawnigan Lake, British Columbia, Canada, 40 years after PCT and fertilization treatments (Li et al., 2018). Li et al. (2022) further demonstrated that CG may also be common in forest stands, using three published long-term silviculture datasets for major tree species across Canada, including lodgepole pine (*Pinus contorta* Douglas) in the MacKey thinning experiment, foothills Alberta, red pine (*Pinus resinosa* Aiton) in commercial thinning experiment of the Petawawa Research Forest, Ontario, and the costal Douglas-fir in the Shawnigan Lake trial. Nevertheless, systematic studies of CG in forestry are still rare, thus limiting the benefit of such exploration for the forest sector, despite significant benefits obtained from CG research results by other industries including livestock, crops, and aquaculture (Li et al., 2021). With the CG as a conceptual framework, however, it is possible to explain and thus predict stand dynamics after a partial mortality. This study is an example of how this framework could provide a mechanistic tool that could benefit forest sector in achieving sustainable forest management.

Methodologically, an experimental approach has been commonly employed to determine whether CG occurred in the experimental systems, and if existed, the type or status of CG. These results can help different industries to benefit from the CG through altering the management regimes (Li et al., 2021). For trees with a long lifespan, however, the experimental approach might not be widely applicable (see a summary in Li et al., 2020). In silvicultural experiments, the normal guidelines defined 10 years after-initial-treatments as appropriate period of observation (e.g., Reukema, 1975 for coastal Douglas fir PCT). However, the results from such a short-term observation might not provide a full picture of how forests might respond to such treatments as demonstrated in Pitt and Lanteigne (2008) and (Li et al., 2018; 2021). Since very few silvicultural experiments last 40 years or beyond, long-term observation results are rarely reported in the literature, simply because they require efforts from at least two continuous generations of professional careers. Therefore, to design and implement field CG experiments and then wait for results might be impractical due to the time and costs restraints. Nevertheless, Li et al. (2022) was able to demonstrate that CG might also be common in forest stands using legacy datasets (30-60 years) at the stand and tree levels of three major tree species across Canada, though these experiments were originally designed for other purposes. Considering that operational decisions in forest management are usually urgent and cannot wait for suitable data to become available, ecological modeling approach can play an important role in assisting decision support (Mangel et al., 2001).

Conceivably, if the CG phenomena commonly occurred in trees and forest stands as demonstrated in Li et al. (2022), one should be able to develop a simulation model, using existing growth-and-yield relationships, to corroborate the CG process within a lifespan of trees and forest stands triggered by thinning. Such a model could help elucidate the mechanism(s) underlying diverse CG patterns and facilitate design of the best strategy to take advantage of the CG phenomena by the forest sector. As plenty of growth and yield relationships are readily available for natural stands across North America and Europe, the first step would be to investigate whether this body of knowledge could be used for forecasting CG consequences. Such an attempt would have at least two meaningful consequences: one would be to produce a tool for predicting CG effect on stand dynamics, and the other would be to confirm that CG is probably an understandable natural process in stand dynamics, rather than a suspicious or out-of-the-ordinary phenomenon. In the current study, we show this attempt is possible through proper utilization of available growth and yield relationships.

The objectives of the current study are two-fold: first, to describe a state-dependent individual tree-based forest growth model called TreeCG, standing for Tree’s Compensatory Growth, representing an alternative modeling approach to predict dynamics of stands after thinning operations; and second, to show how this modeling approach can successfully reproduce the CG patterns observed in long-term silviculture (thinning) experiments. The possible applications of this modeling approach are also briefly discussed.

## Materials and methods

### Simulation model structure and major components

**Figure 2:**
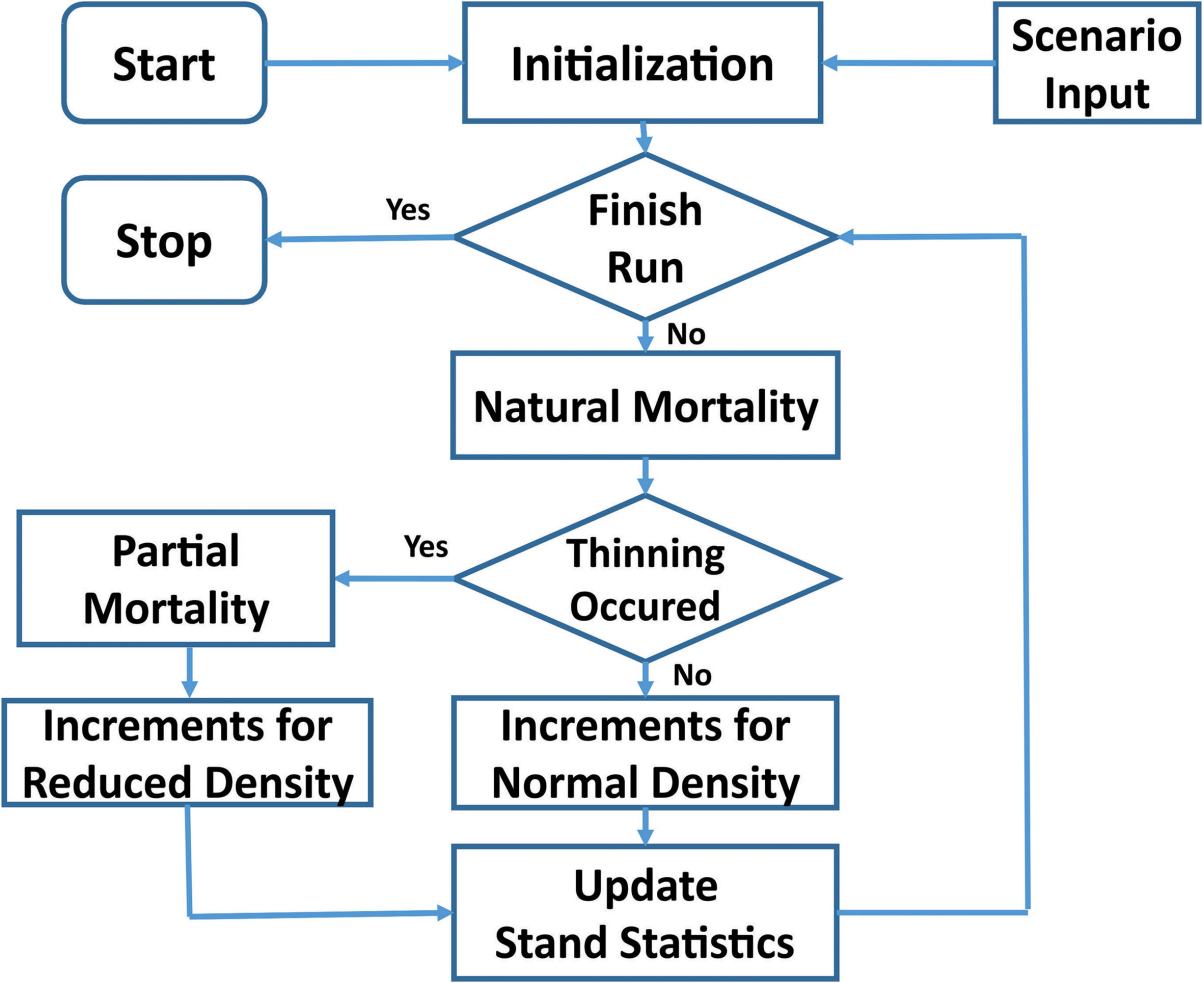
Data flow of the TreeCG simulation model.

The TreeCG forest growth simulation model was used in the current study. Different from age-dependent growth and yield models that aimed at predicting mean stand volume over time under given site index for specific tree species or stand type, the state-dependent TreeCG model simulates the annual increments of diameter at breast height (*DBH*) and height (*H*) of individual trees within a stand, in which the annual increments are determined by the internal state of tree age and external state of resource availability. Therefore, the TreeCG model can capture the detailed responses of trees to changing environment conditions at any given tree age. Results from such a model structure allow estimation of different volume- and value-based assessment of stand productivity. **Figure 2** shows the data flow of our model.

The major components of the model are as follows:

***Initialization***: This component sets up the initial conditions defined in the ***Scenario Input*** including stand density, the years of thinning operation, thinning intensity and method, and the distributions of diameter and height that assigned to each individual tree, thus allowing the program to keep track of the fate of each individual tree over time.

***Natural mortality***: *M_Natural_*, is estimated using the following equation (Botkin et al., 1972; Keane et al., 2011):


(1) $$M_{Natural}=1-\exp(-4/Age_{Max})$$

where *Age_Max_* is the longevity of tree. Lodgepole pine is a pioneer species that is distributed in western Canada and Rocky Mountain area. Kaufmann (1996) reported that the old-growth lodgepole pine trees varied in age from 250 and 296 years with about 31 cm in diameter in the Fraser Experimental Forest near Fraser, Colorado. In current study, 250 was used as the longevity of lodgepole pine.

***Tree growth without thinning***: the variable-density yield tables for natural stands of lodgepole pine in Alberta (Johnstone, 1976) were used to account for the effect of stand density on the relationship between *H* and age, *Age*:


(2) $$\begin{array}{l} \log_{10}H=1.0688-0.00276672\times Age+0.717927\log_{10}Age-\\ 0.0000371084\times Stems-0.132622\log_{10}Stems \end{array}$$

where *H* is height in m, *Stems* is the number of stems no less than 0.6 inch (i.e., 1.524 cm) of *DBH* outside bark per acre and Age was based on years. We converted the units in the formula from British system into metric system, thus enabling the calculation of *H* of each tree at a given stand density.

Tree *DBH* is expressed as a function of *H*, estimated using the softwood equation of Huang (2016):


(3) $$DBH=\{b_{1}/b_{2}\times {\left[1-\exp(-b_{1}\times b_{2}\times (H-1.3)]\right\}}^{1/b_{3}}$$

**Figure 3:**
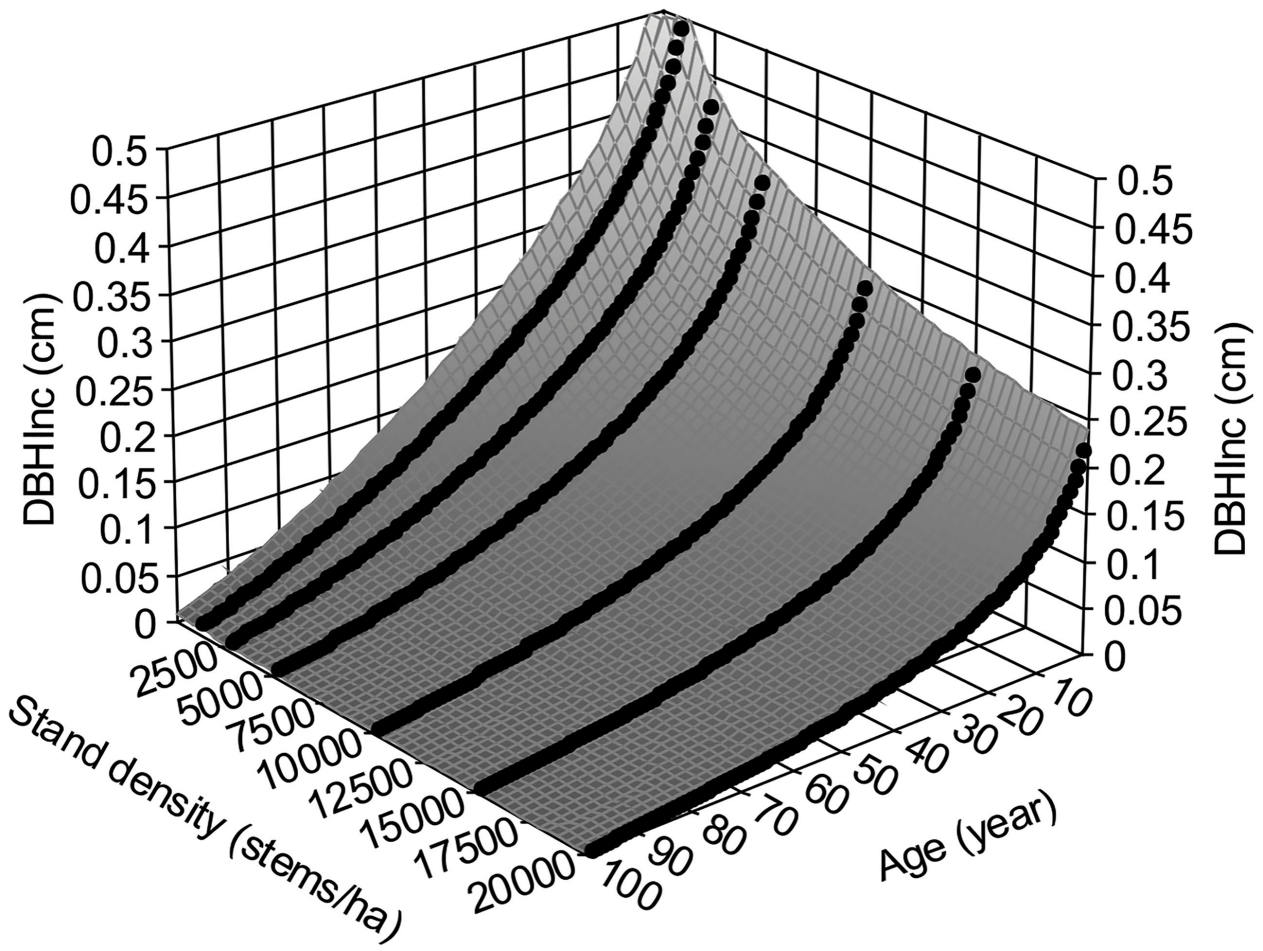
Annual increments of *DBH*, *DBH_Inc_*, for lodgepole pine, at different stand age and stand density based on the variable density yield table of Johnstone (1976).

For lodgepole pine in Alberta, *b_1_* = 2.0584, *b_2_* = -0.0407, and *b_3_* = 1.5310. The annual increment of *DBH*, *DBH_Inc_*, was then calculated as:


(4) $$DBH_{Inc,t}=DBH_{t+1}-DBH_{t}$$

where the subscripts *t* and *t+1* indicate the measured variables at year *t* and *t+1*, respectively. **Figure 3** shows surface plot generated by equation (4), in which the *DBH_Inc_* decreases with increasing tree age and stand density, reflecting the dynamics of tree vigor or vitality. This is consistent with the common observations of faster growth of trees in young stands.

Applying the surface smoothing techniques using the software TableCurve3D (SYSTAT Software Inc., 2002) *DBH_Inc_* at any given age and density could be estimated as:


(5) $$\begin{array}{l} z=a+b\ln(x)+c\ln(y)+d{\left(\ln(x)\right)}^{2}+e{\left(\ln(y)\right)}^{2}+f\ln(x)\ln(y)+\\ g{\left(\ln(x)\right)}^{3}+h{\left(\ln(y)\right)}^{3}+i\ln(x){\left(\ln(y)\right)}^{2}+j{\left(\ln(x)\right)}^{2}\ln(y) \end{array}$$

where *x* is tree age, *y* is stand density, and *z* is *DBH_Inc_*. Parameter *a* = -0.47912889, *b* = -0.09398818, *c* = 0.585319033, *d* = -0.08405214, *e* = -0.09621235, *f* = 0.04071322, *g* = -0.000857125, *h* = 0.004492331, *i* = -0.00377608, and *j* = 0.008262739. The surface fitting has *R^2^* = 0.9942 and *F* = 8652.8217 and *p*< 0.0001.

***Tree growth with thinning***: at the year of thinning, the altered tree *DBH_Inc,new_* can be decomposed as the *DBH_Inc_* of stand density before thinning, *DBH_Inc,natural_*, plus the released growth, *DBH_Inc,natural–new_*, of stand density after thinning:


(6) $$DBH_{Inc,new}(cm)=DBH_{Inc,natural}(cm)+DBH_{Inc,natural-new}(cm)$$

where the subscripts of *natural* and *new* indicate the stand density before and after the thinning operation. To calculate *DBH_Inc,new_* (*cm*), we need to have both *DBH_Inc,natural_* (*cm*) and *DBH_Inc,natural–new_* (*cm*), where *DBH_Inc,natural–new_* (*cm*) is the extra (released) growth of *DBH* induced by the thinning operation. To simplify the *DBH_Inc,natural–new_* (*cm*) calculation, *DBH_Inc,natural_* (*cm*) and *DBH_Inc,new_* (*cm*) are presented in a relative sense (with regarding to an assumed maximal stand density of 20,000 stems/ha for lodgepole pine, and in this case as *RDBH_Inc,natural_* (%) and *RDBH_Inc,new_* (%), respectively:


(7a) $$RDBH_{Inc,natural}(\%)=DBH_{Inc,natural}(cm)/DBH_{Inc,20,000}(cm)\times 100$$

(7b) $$RDBH_{Inc,new}(\%)=DBH_{Incm,new}(cm)/DBH_{Inc,20,000}(cm)\times 100$$

We then calculated *DBH_Inc,new_* (*cm*) as


(8) $$DBH_{Inc,new}(cm)=DBH_{Inc,natural}(cm)\times [1+RDBH_{Inc,natural-new}(\%)]$$

where *RDBH_Inc,natural–new_* (%) is calculated as:


(9) $$RDBH_{Inc,natural-new}(\%)=\frac{RDBH_{Inc,new}(\%)-RDBH_{Inc,natural}(\%)}{RDBH_{Inc,natural}(\%)}\times 100$$

**Figure 4:**
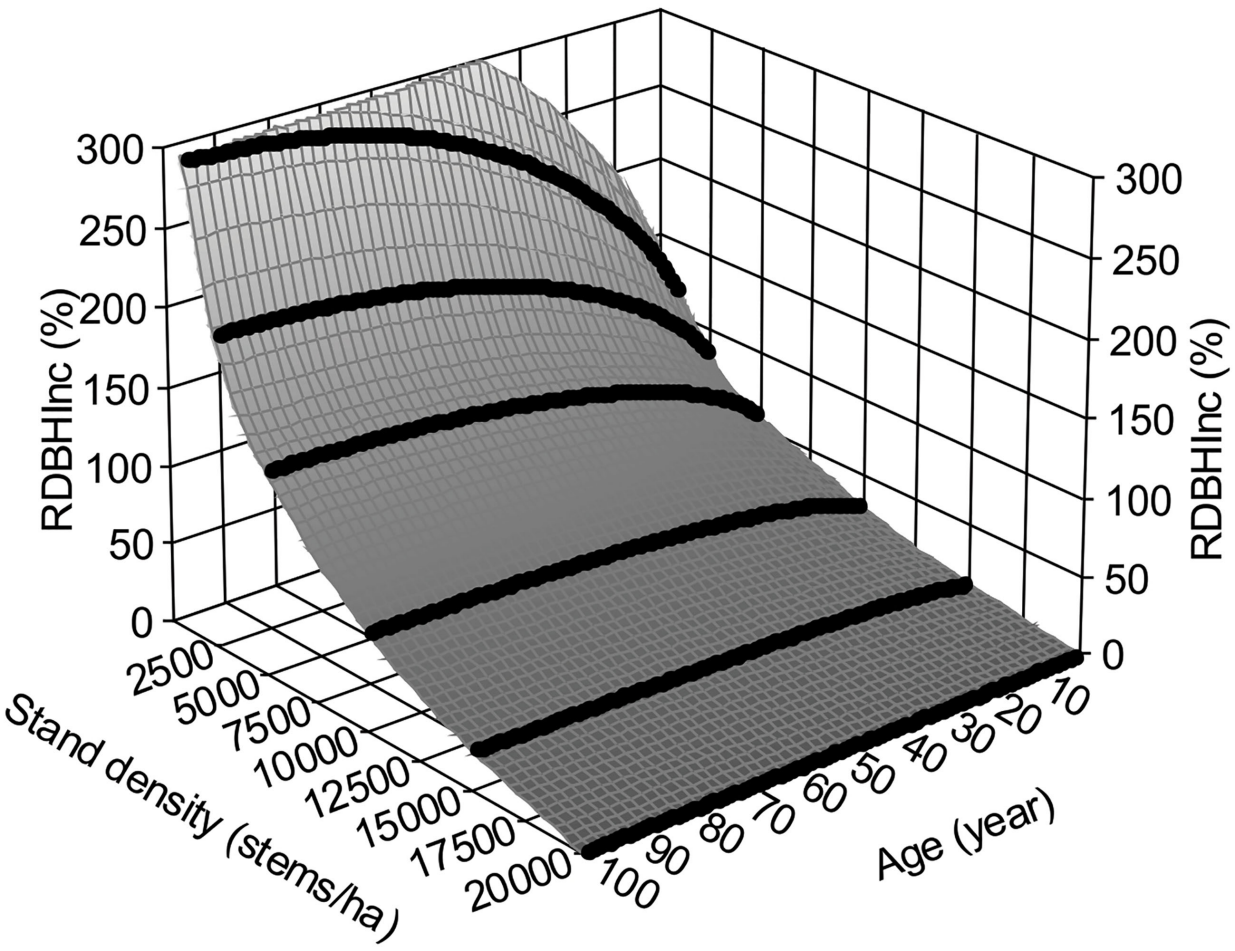
Relative annual *DBH* increment, *RDBH_Inc_* (%), regarding an assumed maximal stand density of 20,000 stems/ha.

With above treatments, the *DBH_Inc,new_* (*cm*) can be directly estimated from the stand densities before and after thinning. The parameters were estimated using the surface smoothing for equation (5), where *x* is tree age, *y* is stand density, and *z* is *DBH_Inc,new_* (*cm*). Results for the numerical simulation results are *a* = 9214.78559, *b* = 2726.639714, *c* = 2258.136021, *d* = -325.333346, *e* = -229.149066, *f* = -259.997371, *g* = 14.89819094, *h* = 8.856809336, *i* = 4.586019568, and *j* = 16.20041778. The surface fitting has *R^2^* = 0.9994 and *F* = 85893.681 with *p*< 0.0001. In other words, *RDBH_Inc,natural–new_* (%) can be obtained by calculating two stand densities, before and after thinning, using the same equation as shown in **Figure 4**.

The surface of *H* after the released growth, corresponding to *DBH_Inc,new_* (*cm*), can then be obtained using the provincial equation for lodgepole pine (Huang et al., 2013):


(10) H=1.3+35.7547/{1+exp[3.8234−1.2824ln(DBH)]}

For clarity, we summarized the set of variables in our model along with their definitions in **Table 1**.

**Table 1:** List of variable definitions.

Variable	Definition
*M_Natural_*	Natural mortality in %
*Age_max_*	Tree longevity in years
*H*	Tree height in m
*Stem*	Number of stems ≥ 0.6 inch (i.e., 1.524 cm) of *DBH* per acre
*DBH*	Diameter at breast height outside bark, in cm
*DBH_t_*	*DBH* measured at year *t*
*DBH_t_*_+1_	*DBH* measured at year *t+1*
*DBH_Inc,natural_* (*cm*)	Annual *DBH* increment, before thinning, in cm
*DBH_Inc,_*_20,000_(*cm*)	Annual *DBH* increment at an assumed maximal stand density of 20,000 stems/ha
*DBH_Inc,new_* (*cm*)	Annual *DBH* increment, after thinning, in cm
*RDBH_Inc,natural_* (%)	Annual *DBH* increment, before thinning, in % relative to assumed maximal stand density of 20,000 stems/ha
*RDBH_Inc,new_* (%)	Annual *DBH* increment, after thinning, in % relative to assumed maximal stand density of 20,000 stems/ha
*RDBH_Inc,natural–new_* (%)	Relative *DBH* annual increment of released growth in %

### Model validation

Since the current version of the TreeCG is generic, our model validation is performed qualitatively in terms of examining whether the model structure and assumptions are reasonable. If the answer was positive, the simulation results should be able to reproduce the diverse patterns of CG in stands after different thinning operations. As an example, we compared the simulated CG patterns qualitatively to what displayed in the Shawnigan Lake long-term silviculture experiments using the relative growth (RG):


(11) $$RG=(Vol_{Thinned}/Vol_{Control})\times 100\%$$

where *Vol_Thinned_* and *Vol_Control_* are the gross volumes for thinned and control stands, respectively. If the model structure and assumptions are reasonable, the simulated RG over time should be able to show the temporal transition from under-compensation to CIE and over-compensation in thinned stands compared to unthinned stands (Li et al., 2018).

### Model simulation experiment

Our model simulations were designed to test the premise that the CG capacity of a tree is a function of internal (age) and external (intensity of thinning, i.e., the level of stimulus/mortality) states. The premise came from the diverse CG patterns observed in different PCT experiments. If this premise were true, the simulation results should show the different responses of a forest stand to different thinning operations. As such, one could expect that existing growth and yield relationships from natural stands could be used to predict the growth trajectories of stands under different thinning operations. Furthermore, one could also expect that an optimal thinning regime or strategy could be identified, in terms of optimal timing and intensity of a thinning operation, for achieving a maximized stand productivity.

The initial stand density was set as 7,500 stems/ha, and the simulation results were evaluated for the total gain over a planning horizon of 100 years, which accounted for the sum of the immediate gain from thinning operation and the gain from the remaining trees at the year 100. Thinning operations were implemented at two different stand ages of 30 (representing an early partial mortality) and 60 (representing a late partial mortality) years old. A control without thinning was also implemented. The partial mortality was represented by the intensity of thinning, which was set as removal of 33% (as a light mortality) and 66% (as a heavy mortality) of total number of trees. If this premise were true, the simulated CG capacity could differ under early and late partial mortality, as well as light and heavy mortality.

The model output includes the stand conditions (*DBH* and *H* of each living tree) at initialization and every 5-year interval until 100 years, and harvested wood at the year of thinning.

### Evaluation of stand productivity

The stand productivity evaluation for the simulated stand dynamics was conducted using the Wood Fibre Value Simulation Model (WFVSM) (Li et al., 2017; Li et al., 2022). As a unified valuation tool, the WFVSM simulates both volume- and value-based indicators of a given tree-based forest inventory, represented by harvested logs segregated into different types of treatment centres for different products, including sawmills, pulp mills, veneer mills, plywood mills, and bio-refineries, as well as used as a source of biomass for heat and electricity, and carbon capture. The calculations of these indicators were primarily based on knowledge from the field of forest engineering such as Briggs (1994), except sawmill recovery, which is based on the simulation results of the Optitek, an industrial sawmill operation software package (FPInnovations, 2014), at possible combinations of *DBH* (at the interval of 1 cm) and *H* (at the interval of 1 m).

Gross volume, merchantable volume, lumber volume recovery, lumber value recovery, and net sawmill value are used in current analysis. The gross volume, *Vol*, was calculated using the lodgepole pine tree volume equation for Alberta (Penner et al., 1997):


(12) $$Vol=\begin{array}{l} 4.421585\times 10^{-5}DBH^{1.926909}H^{1.00304} \end{array}$$

The merchantable volume is the log volume transported to the mill gate. The lumber recovery and all by-products are obtained from the look-up-table tallied from multiple simulations using the Optitek. The total value recovery from sawmill, *Val_Sawmill_*, can be calculated as


(13) $$Val_{Sawmill}=Val_{Lumber}+Val_{Chips}+Val_{Sawdust}+Val_{Shaving}+Val_{Bark}$$

where *Val_Lumber_*, *Val_Chips_*, *Val_Sawdust_*, *Val_Shaving_*, and *Val_Bark_* are the value recoveries from lumber, chips, sawdust, shavings, and bark, respectively. The lumber sale price was defined as $400 per thousand board feet (MBF), chips sale of $140 per Metric Tons (MT), sawdust price of $25/MT, shaving price of $15/MT, bark price of $10/MT, and the average wood density was set to 400kg/m^3^. The costs in sawmill operations are also included in the Optitek simulations, which are the fixed costs of $15/m^3^ lumber recovery, planing costs of $25/MBF, drying cost of $15/MBF, and sawing cost of $60/MBF. These values employed were taken from the default set in the Optitek software package reflecting the mean market situation of early 2000s in North America, and the mean operational costs from a large number of Canadian sawmills at the time. The values used in this investigation serve as a standard of comparing simulation results under different thinning operations. The Optitek provides a flexibility of allowing users to define their own values based on their specific mill machine configuration and fluctuating commodity market.

## Results

### Simulated forest growth pattern

A direct method of exploring the outcome of a stand dynamics model is to examine the long-term stand growth trajectories under different thinning scenarios. When no thinning operation is applied, the modeled stand growth trajectory will constitute a forest growth pattern under the control scenario, equivalent to natural stand condition. This trajectory can serve as a baseline case to compare with each of simulated growth trajectories under different partial mortalities caused by prescribed thinning operations. An over-compensation can be indicated when the stand growth trajectory is higher than the baseline case. An under-compensation will be denoted when the stand growth trajectory is lower than the baseline case. An exact compensation, or CIE can be called when the stand growth trajectory ends up at some point being equal to the baseline case (see illustration in **Figure 1**).

**Figure 5:**
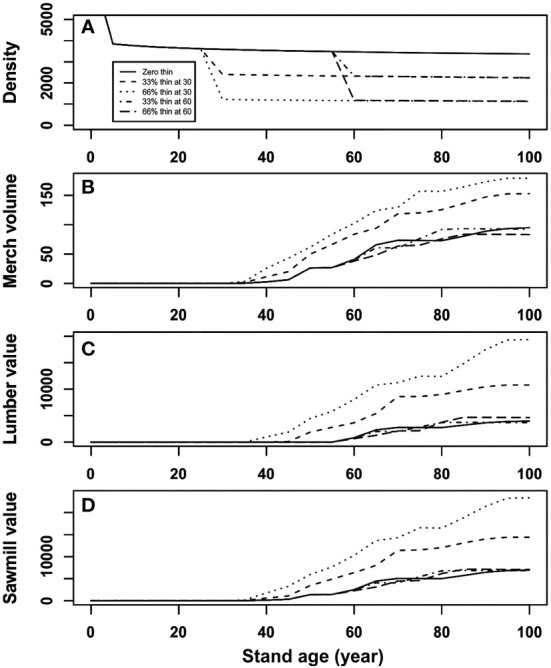
Simulation results of **(A)** stand density (stems/ha), **(B)** merchantable volume (m^3^/ha), **(C)** value recovery of lumber ($/ha), and **(D)** total value recovery of sawmill ($/ha) of initial stand density of 7,500 stems/ha under different thinning intensities for a hypothetical stand of lodgepole pine at age 30 and 60 years.

The results of our simulation were expressed throughout a graphical representation that covered a planning period of 100 years (**Figure 5**). Stand density decreased exponentially at the early stand development that is determined by the natural mortality function (equation (1)), and then decreased proportionally at the year of thinning operation according to the intensity of partial mortality (**Figure 5A**). Moreover, our results also show that final merchantable volume after early partial mortality can exceed that from control, i.e., overcompensation, and a heavier mortality could result in a higher merchantable volume than that from a lighter mortality (**Figure 5B**). However, such a trend may not always be the case when a late partial mortality is experienced, which resulted in either under-compensation or CIE. In other words, both thinning intensity and timing can result in different stand productivity at the end of simulation. In addition, the evolution of the lumber and sawmill value (Figs. 5c and 5d) could suggest an increase when value recovery occurred from the merchantable volume, which is probably because of the increased percentage of large dimension lumber products.

The results suggest that the stand’s capability for compensation could be higher at a younger age than that at an elder age, and heavier thinning could result in a higher compensation capability than that from a lighter thinning. The simulation results also suggest that a partial harvest performed at age 60 might be too late with very little or no benefit to be expected from the CG phenomenon. This is probably true because age 60 is usually the minimal stand age of conventional harvest in many jurisdictions based upon decelerating growth.

### Simulated total gains

Forest managers usually take a certain planning horizon for evaluating the consequences of a given management operation. The planning horizon could vary depending on the purpose of planning. For example, a typical forest harvest planning process could have multiple 20-year planning periods with a total of 200 years as the strategic planning horizon for the province of Manitoba, Canada (Li et al., 2011). In the current study, we explored the consequences of a thinning operation during a planning horizon of 100 years, with every 5-year as an output period. This enables evaluation of changing stand value over the planning horizon.

To evaluate the consequences of different thinning operations, we compared several indicators of total gain at the end of a planning horizon of 100 years for the operations as showed in **Table 2**. In the case of early thinning, there is no immediate benefit from the operations, but the final harvest could be significantly higher than that from the unthinned stand. Late thinning can have some immediate benefit from operations (11.308 and 23.242 m^3^/ha merchantable volumes from 33% and 66% removal of surviving trees, respectively); however, they could not make up the lost in final harvest at the end of planning horizon for the total merchantable volume, although the value recoveries could still be higher than that from unthinned stands probably due to the large dimension of sawlogs. Simulation results showed that under a late thinning regime, thinned stands would eventually (> 15 years) outperform unthinned ones after an initial underperformance.

**Table 2:** Simulated gains from different timings and intensities of thinning over 100 years.

Treatment	Items	From thinning	From final harvest	Total
**Control**	**Merchantable volume (m^3^/ha)**		94.841	94.841
	**Lumber vale recovery ($/ha)**		3,958.86	3,958.86
	**Sawmill value recovery ($/ha)**		6,981.77	6,981.77
**1/3 mortality at 30 years**	**Merchantable volume (m^3^/ha)**		152.8	152.8
	**Lumber vale recovery ($/ha)**		10,792.20	10,792.20
	**Sawmill value recovery ($/ha)**		14,408.52	14,408.52
**1/3 mortality at 60 years**	**Merchantable volume (m^3^/ha)**	11.308	80.687	91.995
	**Lumber vale recovery ($/ha)**	144.13	3,521.51	3,665.64
	**Sawmill value recovery ($/ha)**	658.497	6,150.306	6,808.803
**2/3 mortality at 30 years**	**Merchantable volume (m^3^/ha)**		178.869	178.869
	**Lumber vale recovery ($/ha)**		19,383.74	19,383.74
	**Sawmill value recovery ($/ha)**		23,350.73	23,350.73
**2/3 mortality at 60 years**	**Merchantable volume (m^3^/ha)**	23.242	59.722	82.964
	**Lumber vale recovery ($/ha)**	326.5	4,282.64	4,609.14
	**Sawmill value recovery ($/ha)**	1,367.36	5,729.17	7,096.53

These results suggest that at the earlier stage of stand development, trees might have a higher capacity of CG due to increased vigor of surviving trees. The capacity of CG could reduce with aging trees. Therefore, to take full advantage of CG capacity in enhancing long-term stand productivity, the thinning should not be performed too late (e.g., before 60 years of stand age in current planning scenario) in the process of stand development.

### Model validation

**Figure 6:**
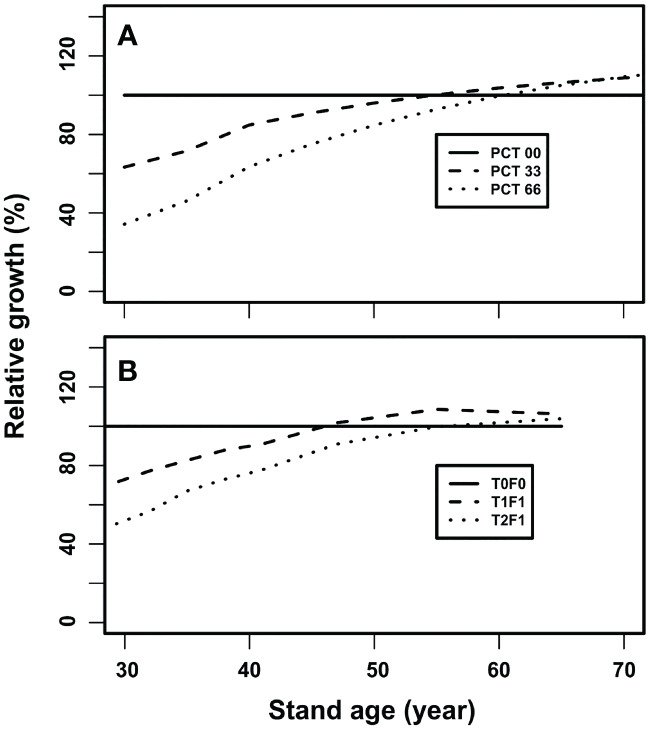
The stand growth trajectories after experiencing a thinning operation: **(A)** simulated growth trajectories after experiencing a PCT at age 30, and **(B)** observed growth trajectories after PCT at age 24 based on the PCT and fertilization (224 kg N/ha) treatments at the Shawnigan Lake trial, BC Canada.

A qualitative model validation was conducted through comparing the simulated forest growth patterns to a long-term silvicultural experiment, which was a 40-year trial of PCT and fertilization on the coastal Douglas-fir near the Shawnigan Lake, British Columbia (Crown and Brett, 1975). The focus of the comparison is not *via* matching of absolute values, because the relationships employed in our simulation model were not specifically from the coastal Douglas-fir forests. The dataset from the Shawnigan Lake trial contains multiple repeated measurements for a combination of three levels of PCT and three levels of fertilization treatments. As reported in Li et al. (2018), the diverse stand growth patterns can be expressed by growth relative to the control in percentage. In the current model validation, the relative growth is used to compare the CG growth patterns as shown in **Figure 6**.

As a longitudinal process, one can follow stand gross volume after experiencing an early thinning at year 30 under three levels of intensity (i.e., 0, 33%, and 66% removal for the initial stand density of 3,950 stems/ha). Here, partial mortality first reduced gross stand volume but then the stand gross volume gradually catches-up with that of the control stand, and eventually exceeds the control and generates over-compensation (**Figure 6A**). The heavier thinning (66% mortality) resulted in higher over-compensation than that of the lighter thinning (33% mortality) (**Figure 6A**). The trends displayed in this series of simulations appeared consistent with observed by the 40-year’s measurements from the Shawnigan Lake trial (**Figure 6B**). This indicates that our simulation results are validated qualitatively by the observations from this long-term PCT and fertilization experiment.

Note that only the simulation results between age 30 (the year the thinning operations are conducted) and 70 are plotted, because the observations from the Shawnigan Lake trial cover 40 years only as the treatments were started at age 24 and measured for 40 years.

Our results suggested that short-term (10-year) observations defined by Reukema (1975) after PCT, will generate under-compensation (**Figure 6**). The over-compensation can only be observed in long-term observations such as 40-year after the PCT treatments. This is because slow growing trees need sufficient time for the treated stands to compensate the lost volume at the treatments. This confirmed that the sufficient condition for over-compensation to occur is having enough time (Li et al., 2018).

## Discussion

Our results confirmed the premise that a simulation model based on existing growth and yield relationships, can reproduce diverse CG patterns observed in long-term thinning experiments. In this section, we discuss our CG research approach in general, examine the logical consequences of thinning, and briefly go over the potential management applications of our model TreeCG.

### CG research approach

Management of forest growth is one of the major components in modern forest management. It aims at promoting productive forestry, in terms of proactively seeking ways of enhancing forest productivity (Li et al., 2020). In other words, a major concern is how management operations might enhance forest productivity. To reach this goal, improved understanding on the dynamics of managed stands appears essential. Existing forest growth and yield models characterize the growth responses of forest stands under natural conditions including some fluctuations caused by some random events (e.g., Martin, 1991; Huang et al., 2001). However, it has not been thoroughly examined as to whether these relationships also apply to managed stands, probably due to insufficient data sources. Nevertheless, some studies revealed that they are probably not the same. This was evidenced in Huuskonen and Hynynen (2006), who found that the diameter increment of trees after PCT can be characterized by a function of dominant height, initial stand density and the actual stem number of the PCT treated growing stock, and the regeneration method. Recently, Burkhart and Yang (2022) found that the carrying capacity, an indicator of productivity, in intensively managed plantation plots of loblolly pine (*Pinus taeda*) was significantly higher than that of non-intensively managed plantation plots, which suggested that stand growth trajectories in the two types of plantations were different.

**Figure 7:**
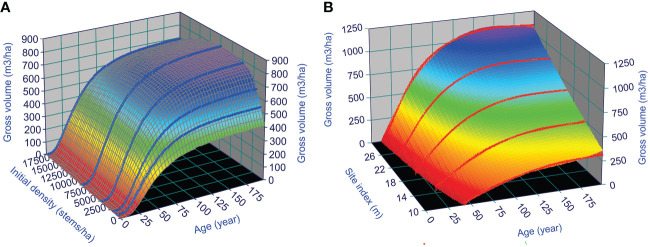
GYPSY model predicted surfaces of lodgepole pine yield in AB, Canada: under different **(A)** initial stand densities when site index is 30 m, and **(B)** site index when initial stand density is 7,500 stems/ha.

Existing growth and yield relationships generally assume that the observed productivity of a forest stand results from the optimal utilization of space and nutrients of the trees living in the stand (Li et al., 2020). Thus, the growth and yield models of natural stands should represent the optimal stand productivity. This can serve as a starting point for understanding the dynamics of managed stands, such as the CG phenomenon after thinning. As an example, such yield surfaces of lodgepole pine can be illustrated in **Figure 7**, by using GYPSY model (Huang et al., 2001; Huang et al., 2009) in Alberta (AB), Canada: the stand yield surface under different initial stand densities over stand age when site index is 30 m (**Figure 7A**), and under different site indices over stand age when initial stand density is fixed as 7,500 stems/ha (**Figure 7B**). These surfaces should allow practitioners to estimate the stand productivity of the species at any given stand age, site index, and initial stand density.

When a thinning operation is conducted at a specific stand age, one could assume that the stand productivity would switch from the stand growth trajectory from the initial stand density to the stand growth trajectory indicated by the stand density after the thinning operation at the specific stand age. Though this assumption could be too rough possibly due to delay for the stand to detect the new level of space and nutrients availability under the new stand density, it provides a possible way of predicting stand productivity after the thinning operation, using the information contained in growth and yield curves for natural stands.

A challenge in implementing this switch is to determine the corresponding initial stand density for the trajectory with stand density after the thinning operation at the specific stand age. Such a challenge was also appeared in studying population dynamics of mountain pine beetle (*Dendroctonus ponderosae* Hopkins) (MPB) after a management operation, in which the challenge is the estimation of population density after the operation. Safranyik et al. (1999) developed a MPB population dynamics model at a spatial scale of one hectare, to forecast the population dynamics with a flexible initial population density. However, the population reduction from a management operation will require the trajectory of the population dynamics to be adjusted due to the reduced population density at the time of the operation. Therefore, researchers had to perform two separate runs of the model: one for population dynamics before the operation under the initial population density, and the other for population dynamics after the operation under the reduced population density. Due to the stochastic nature of the model, it appeared difficult to estimate the initial population density under which the population density after the operation could be exactly harmonized, and multiple runs of guessed initial population densities might be needed to match accurately the population density after the operation.

This guessing of initial population density is similar to the challenge of modelling the stand growth trajectory after a thinning operation. Despite the fact that stand density after the thinning can be relatively easy to estimate, the new trajectory at different timings could still present a challenge in identifying the corresponding initial stand density under which the stand density will be the same as the reduced stand density right after the operation. This means multiple runs might be needed to “guess” the right growth trajectory for after the thinning operation. TreeCG model resolved this issue by developing a new algorithm described in the methods section.

Most existing forest growth and yield models are analytical, with a premise that once the specific tree species or stand type and site index are known, stand yield curve of the natural stand can be determined (e.g., King, 1966). This fixed shape of stand yield curve for natural stands facilitates straightforward applications by practitioners but compromises the flexibility of capturing changes between years. For managed stands, such a fixed shape of stand yield curve might not always apply because significant disturbances such as both catastrophic fire events and thinning operations could reduce biomass or stand density and thus alter the stand yield curve dramatically. To capture such changes between years, a simulation approach would likely perform better than an analytical approach, because it usually handles better the complicated system dynamics that are difficult to represent by a single analytical model. In the state-dependent forest growth model TreeCG, the dynamics of stand volume consist of before and after thinning stages, and the stand volume at any given year is the sum of volumes of individual trees within the stand, calculated yearly using its annual increments of *DBH* and *H*. Since the simulation results will reassemble the dynamics of natural stands under different stand density and site index conditions, the TreeCG model can simulate the dynamics of both natural and managed stands.

The CG phenomenon has been well-known to animal and annual plant researchers, but less familiar to foresters. Application of the CG concept to tree and forest biology is an example of how forestry can benefit from the principles and results of general biology and ecology. The modeling approach presented in current study could also be applied to different industries or fields to take advantages of CG. Theoretically, the modeling approach could be complementary to the experimental approach commonly employed in functional trials (Zirbel et al., 2017; Hanisch et al., 2020). It speeds up our understanding about functional plant ecology.

### Logical consequences from thinning operations

Research results from thinning and stand density management are the primary driver for our understanding CG phenomenon in forests. Combined with initial spacing, thinning is widely used for stand density management (e.g., Long, 1985). It results in a partial mortality to the stand (Bose et al., 2014), and also altered stand structure (Pamerleau-Couture et al., 2015), radial growth response (Montoro Girona et al., 2016), wood quality (Lemay et al., 2018), and mortality (Montoro Girona et al., 2019) and windstorm after partial harvest (Lavoie et al., 2012). Conceptually, if the stand does not respond to the partial mortality, the surviving trees will continue their normal growth trajectories and thus no compensation would occur, which result in the freed nutrients and space from dead trees left unused. However, this is unlikely owing to the inter-tree competition. If partial mortality could be seen as a stimulus to the normal growth of the stand, it could trigger a CG for the remaining trees in the stand. Obviously, compensation will not occur in dead trees, however, CG could happen at the stand level such that the surviving trees grow faster than normal. This is achieved by utilizing the extra nutrients and space freed from the dead trees, as indicated by widely observations from PCT experiments (e.g., Bose et al., 2018). As we know, each thinning regime is a combination of the timing and intensity of a thinning operation. If the response of a stand to partial mortality would differ under different thinning regimes, one could expect diverse response curves or growth trajectories of the stand after thinning operations. Thus, the challenge is to identify the best combination of timing and intensity of a thinning operation that can lead to the maximized stand productivity (Li et al., 2020). This appears consistent with the goal of silviculture experiments (e.g., Zeide, 2008).

**Figure 8:**
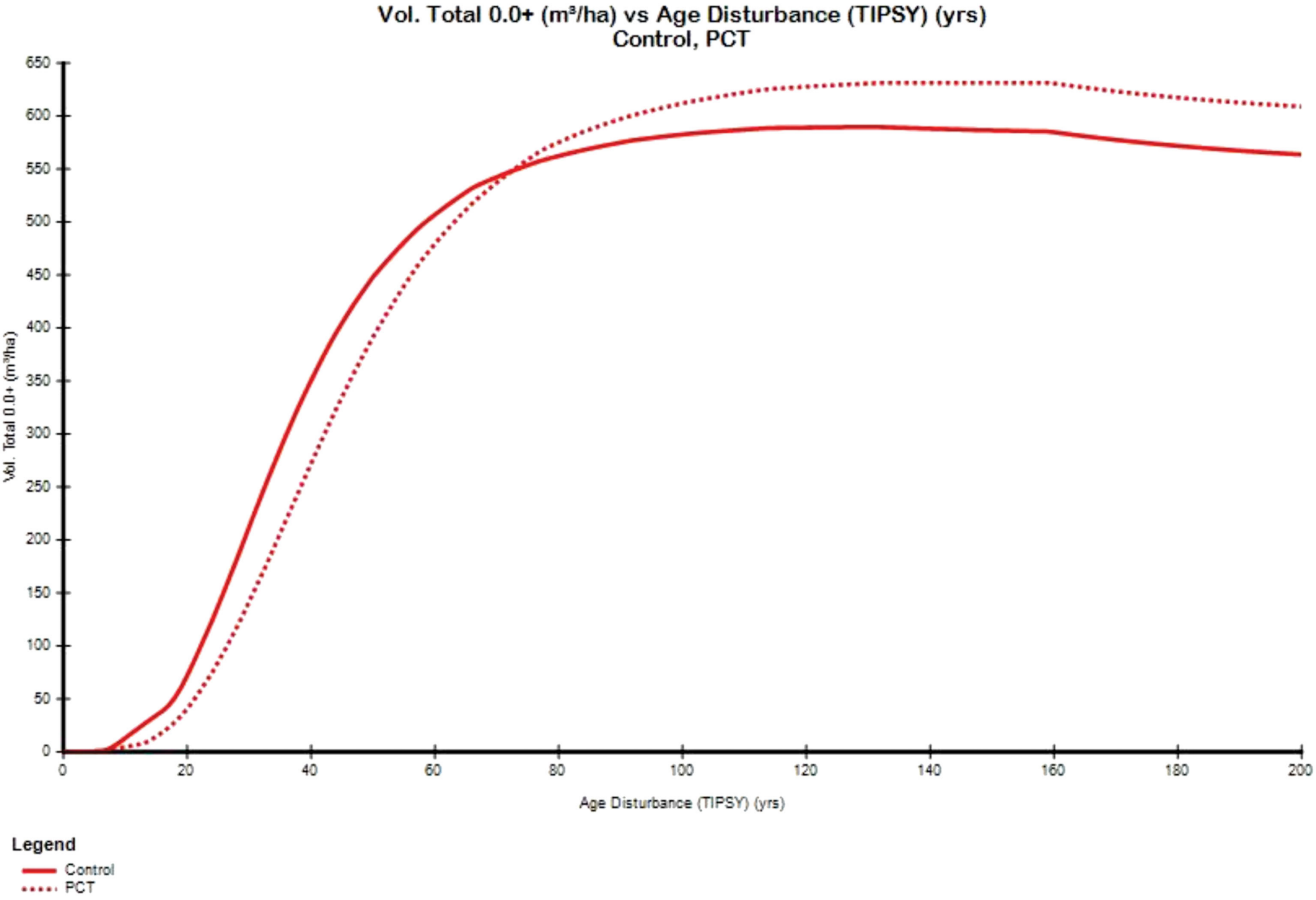
A CG process simulated by using the TIPSY 4.4 through comparing the stand growth curves (PCTed vs. control) in a planted lodgepole pine stand with a site index of 24 m.

Redistribution of resources (including space and nutrients) from dead trees to surviving trees has been implemented in some process-based models. For example, the Tree And Stand Simulator (TASS) model (Mitchell, 1975) demonstrated that PCT and fertilization could lead to overcompensation in terms of basal area as a result of the redistribution of space and light. Unfortunately, this potential had been largely ignored until very recently a PCT experiment conducted using the Table Interpolation Program for Stand Yields (TIPSY, a “meta-model” software program giving electronic access to a vast database of yield tables produced by TASS) showed that PCT could result in a full CG process in stand growth trajectory including overcompensation (**Figure 8**), in which a CG process is triggered by a PCT at year 10, and over time experiencing under compensation up to year 75, exact compensation at year 76, and overcompensation after year 77. Though 65 years are needed for the catch-up in stand gross volume in the PCTed site, that is about a quater of lodgepole pine’s lifespan, which might not be too slow compared with most short lifespan species proportionally. Considering the importance of nutrients in tree growth, a reasonable inference is that the real CG could probably be even more significant and clear than simulated by TASS-TIPSY model.

The TreeCG model provides a possible way of speeding-up the understanding of CG mechanism through the exploration of the model behaviour, or logical consequences from different thinning operations. This exploration can be performed through carefully designed multiple simulations, or model experiments. For example, effect of different thinning regimes on the stand productivity could be conducted by different timing and intensity of thinning, and the simulation results could be evaluated by a unified procedure of volume and value-based inventory assessment, i.e., by running the WFVSM in our case. The WFVSM can provide fast assessment to measured silviculture results of an experiment with the unified procedure, despite its absolute values of indicators could vary slightly in different settings of product treatment centres. In other words, the logical consequences of different thinning prescriptions on stand productivity can be investigated through combination of using a simulation tool of TreeCG and a valuation tool like WFVSM.

### Management applications

**Figure 9:**
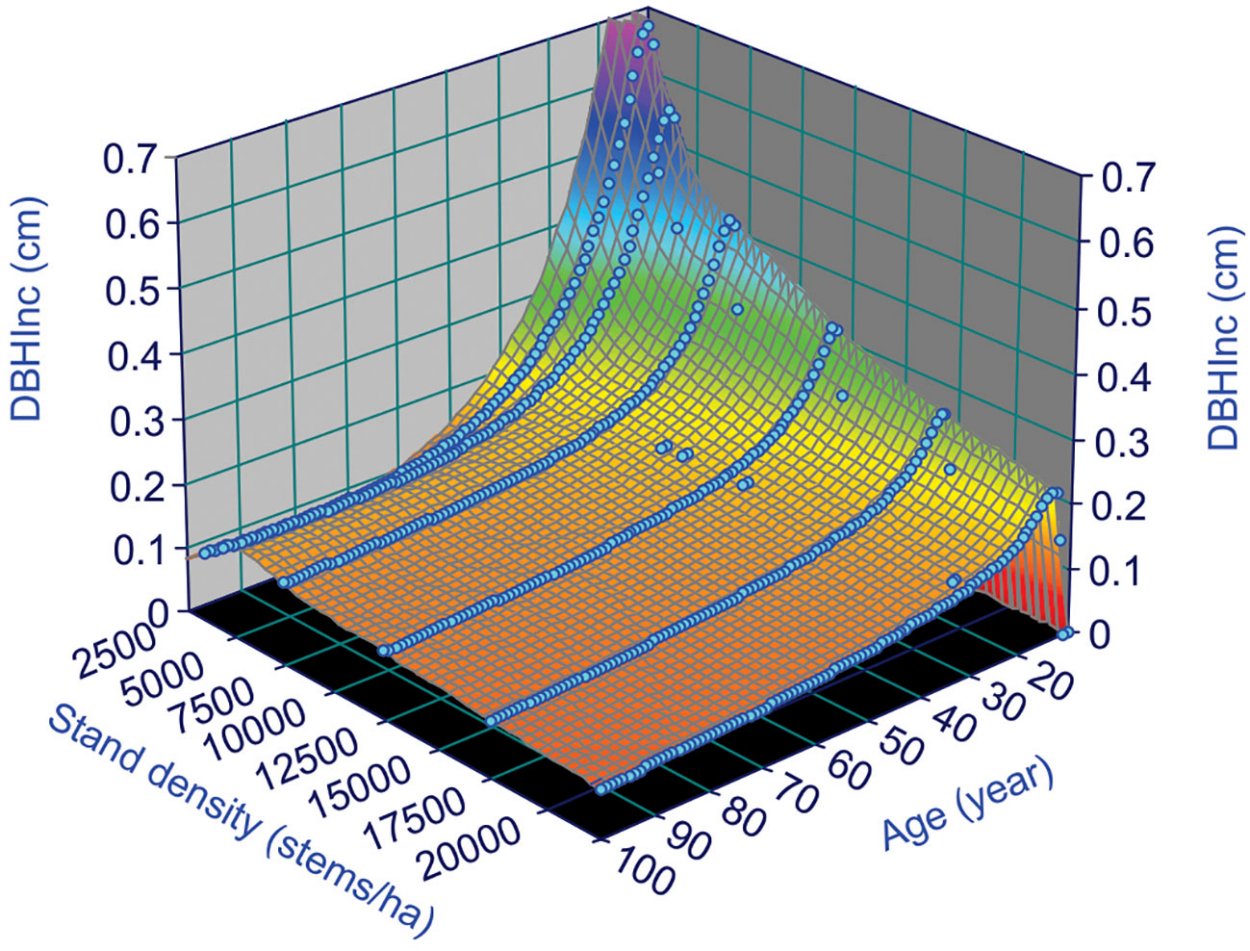
Annual increments of *DBH*, *DBHInc*, of lodgepole pine predicted by GYPSY model in Alberta, Canada, at the site index of 20 m and different stand age and stand density.

Our generic TreeCG model can be refined using local growth and yield relationships for addressing specific regional issues. For example, the most recent lodgepole pine stand growth projection (**Figure 9**) using the GYPSY model (Huang et al., 2001; Huang et al., 2009) with more specific on-site index could replace the surface presented in **Figure 3**, for the calibration to address specific issues under investigation in Alberta. Similar approaches can be applied to other jurisdictions such as British Columbia using the relationships built-in the Variable Density Yield Projection (VDYP) model for natural stands (British Columbia Ministry of Forests, Lands, Natural Resource Operations and Rural Development, 2019).

Calibrated growth and yield models can help addressing practical issues such as:

- Estimation of released growth for crop trees;
- Identification of optimal prescriptions of thinning operations for maximized stand productivity;
- Exploration of optimal spacing for plantation programs;
- Determination of possible elevation of regional annual allowable cut (AAC) through enhanced stand productivity for mitigating the wood supply shortfall;
- Enhancement of carbon capture through forest management for improving regional carbon budget;
- Reduction of fuel accumulation through partial harvest for decreasing fire risk;
- Improvement of biofuel and bioeconomy through increased feedstock for producing heat and electricity.

Since there has been no reported evidence showing that trees are capable of distinguishing the causes of partial mortality, the TreeCG can be expanded to include partial mortality induced by other disturbances such as fire, insect outbreaks, and windstorm, etc. to capture the corresponding responses of forests.

The TreeCG model can help when estimating the growth trajectories influenced by the disturbance regimes altered by climate change. Like most existing growth and yield models, however, the TreeCG model might be technically unsuitable to address potential climate change impact on the growth trajectories of trees and stands directly, simply because it does not respond to changes in climate variables. It would be better to have a climate sensitive growth and yield model through an alternative approach, such as from a life-history perspective that might be more suitable because it takes adaptation of trees to changes in the probability distributions of climate variables and disturbances into account, with hybrids of existing growth and yield relationships. We will describe this approach in a separate manuscript.

## Conclusions and research recommendations

A state-dependent TreeCG model based on the CG conceptual framework was able to reproduce the CG patterns observed in the Shawnigan Lake long-term PCT and fertilization experiment. The new algorithm of simulating CG process reduced the computing demand and simplified the calculation of annual growth increments for individual trees over time. Our results suggested that stand growth trajectories could be altered considerably by different timing and intensity of thinning operations. They explained the diverse CG patterns observed in different PCT experiments. Our model TreeCG can contribute to the understanding of growth trajectories of stands after a partial mortality and could be utilized to address many questions in theory and applications.

## Data availability statement

Permission to use the Shawnigan Lake dataset in this study was obtained from the data provider. Requests to access this dataset should be directed to Cosmin Filipescu of the Canadian Forest Service.

## Author contributions

CL, HB, and SH: model design, implementation, model experiment, data analysis, and draft writing. CL, HB, SH, BR, RL, WX, and YC: review, revision, and editing. All authors contributed to the article and approved the submitted version.
